# Recognizing Distress in Cancer Patients in Day Hospital, by Trained Nurses vs. Non-Trained Nurses: A Pilot Study

**DOI:** 10.3390/healthcare12242498

**Published:** 2024-12-11

**Authors:** Laura Iacorossi, Chiara Falcicchio, Francesca Gambalunga, Emanuela Taraborelli, Gabriella Maggi, Irene Terrenato, Fabrizio Petrone, Anita Caruso, Maria Perrone

**Affiliations:** 1 Department of Life, Health and Health Professions Sciences, Link Campus University, 00165 Rome, Italy; l.iacorossi@unilink.it; 2 Psychology Unit, IRCCS “Regina Elena” National Cancer Institute, Via Elio Chianesi, 53, 00144 Rome, Italy; chiara.falcicchio@ifo.it (C.F.); gabriella.maggi@ifo.it (G.M.); anita.caruso@ifo.it (A.C.); maria.perrone@ifo.it (M.P.); 3 Professional Health Care Services Department, University Hospital “Policlinico Umberto I”, 00161 Rome, Italy; 4 IRCCS “Regina Elena” National Cancer Institute, Via Elio Chianesi, 53, 00144 Rome, Italy; emanuela.taraborelli@ifo.it; 5 CTC and Biostatistics and Bioinformatics Unit—Scientific Direction, IRCCS “Regina Elena” National Cancer Institute, Via Elio Chianesi, 53, 00144 Rome, Italy; irene.terrenato@ifo.it; 6 Health Professions Unit, IRCCS “Regina Elena” National Cancer Institute, Via Elio Chianesi, 53, 00144 Rome, Italy; fabrizio.petrone@ifo.it

**Keywords:** distress, nurse, pilot study, training, cancer patient, advanced competence

## Abstract

**Background**: Psychological distress impacts 35–40% of cancer patients, significantly affecting their quality of life, treatment adherence, and relationships with healthcare professionals. Given this, there is a critical need to enhance nursing competencies to effectively monitor and address psychological distress. Previous studies have highlighted discrepancies in capabilities based on nurses’ training status, emphasizing trained nurses’ critical role in providing appropriate psycho–social referrals. **Objective**: To evaluate the impact that trained nurses have on the detection of distress and the timely referral of patients for a psycho–oncology consultation. **Methods**: A blinded, random, descriptive, monocentric pilot study was conducted. The participants were adult patients in Day Hospital 1 of the National Cancer Institute Regina Elena, Rome, irrespective of illness stage. Tools used included a socio-demographic and clinical data form, distress thermometer (DT), and visual analogic scale (VAS). Patients were randomly divided into two groups: Group A, where questionnaires were administered by trained nurses, and Group B, where non-trained nurses administered questionnaires. Nurses indicated whether patients needed a psycho–oncology consultation. All patients were then seen by a psycho–oncology specialist to determine whether the nurse’s referral was appropriate. Patients and psycho–oncologists were all unaware of the nurses’ training status. The effectiveness of the training was measured by the degree of agreement between evaluators. **Results**: This study involved 20 patients and four nurses. The average DT score was 5, mainly related to physical and emotional problems. Agreement between evaluators was higher in the trained nurses’ group. **Conclusions**: Specific training on DT enabled nurses to acquire advanced skills to accurately refer patients for psychological consultations.

## 1. Introduction

Cancer can significantly impact all aspects of the lives of patients and their families, including psychological, sexual, and family relations [1]. The most frequent emotional reaction in cancer patients is psychological distress. Cancer diagnosis can become a high and chronic stressor and thus contribute to persistent psychological distress in patients with cancer [2]. On the one hand, cancer patients with psychological distress are more likely to be diagnosed with psychological disorders [3]. On the other hand, psychological distress is associated with increased cancer incidence [4] and worse prognosis [3]. Distress has been defined as “an unpleasant emotional experience that can interfere with how a patient deals with cancer itself, with its physical symptoms and with the therapy”; distress can be characterized by normal feelings of vulnerability, sadness, and fear or by symptoms that are more indicative of a psycho–pathological problem [5,6]. Between 30 and 40% of cancer patients throughout the different phases of the disease experience various levels of distress, while approximately 15–20% show symptoms that are insufficient to diagnose a full-blown psychological disorder but which do have consequences for the patient’s health and social relationships [7,8]. This psychological framework presents itself as an independent factor worsening quality of life [9], increasing the risk of psychological distress, reducing treatment adherence, altering the doctor–patient relationship, prolonging recovery and hospitalization times, reducing the biological effectiveness of therapy, decreasing survival rates, and increasing the risk of disease recurrence [10,11]. To quickly identify psychological distress, the use of rapid tools is recommended. The guidelines developed by the National Comprehensive Cancer Network (NCCN) for Distress Management [6] are particularly appropriate. The distress thermometer, the tool used in the pilot study, has been validated and applied to all cancer patients in all stages of the disease and in different cultural contexts [12]. Although the value of routine screening for distress has been widely documented [13], numerous studies have highlighted the difficulties that healthcare professionals have in detecting psychological problems and distress, often due to a lack of specific training on these aspects [14,15]. Among healthcare workers, nurses play a fundamental role in supporting the emotional well-being of patients. In fact, in addition to clinical skills, which include administering medications, monitoring vital signs, wound management, etc., nurses must possess communication and empathy skills. A crucial aspect of nursing skills is the ability to build a trustful relationship with patients, listening to and addressing their emotional needs, as nurses are always in close contact with patients throughout the various stages of the diagnostic and therapeutic process. Furthermore, for many patients, it may be easier to relate to a nurse rather than to other healthcare workers. In this regard, the literature emphasizes the role of the nurse in monitoring distress [16] and in interviews aimed at identifying psychic discomfort [17]. When distress is detected, sending the patient for a psychosocial consultation is often insufficient [18]. It is important for a healthcare unit treating cancer to be trained in distress, to enable timely recognition and management and to be able to refer patients for psycho–oncological consultation when necessary [1]. Considering that nurses play a crucial role in improving the health of patients, not only regarding disease, physical conditions, and treatment but also in terms of emotional, mental, and social well-being [19], it is necessary to train the right healthcare professionals [1,20,21]. Although the value of routine distress screening has been widely documented [13], there is still a lack of data regarding the skills needed to administer the screening, as well as an understanding of the complexity of its implementation in different clinical contexts. The objective of this study was to assess the impact of the training of nursing staff on the detection of distress and the timely referral of patients for psycho–oncological consultation. This study is intended as a preliminary investigation, laying the groundwork for future research in this area.

## 2. Materials and Methods

### 2.1. Design

We conducted a monocentric, randomized, blinded pilot study.

### 2.2. Population and Setting

The sample was made up of patients recruited at Day Hospital 1 (DH1) of the IFO (Istituti Fisioterapici Ospitalieri), National Institute for Cancer Regina Elena, Rome (IRE), and nursing staff working in DH1. The criteria for inclusion of patients were being ≥18 years of age, having a diagnosis of histologically confirmed cancer, undergoing chemotherapy treatment at DH1 of the aforementioned facility, being available for psychological counseling, and being willing to adhere to the study protocol (informed written consent). Patients with cognitive impairment or pathological conditions that might have been an obstacle to their active participation in the study were excluded. The inclusion criteria for nurses were being employed at the DH1, being available to participate in a one-month training course, and having not participated in any training courses on communication skills in the previous two years. The random assignment of nurses to a training group and not sharing information with the patients made it possible to evaluate whether there were any behavioral biases among the nurses during the study, as well as avoiding a scenario in which knowledge of this information could influence the patients during the interviews conducted by the nurses.

### 2.3. The Measurement Tools

The tools used were the following: a form for the collection of patients’ socio-demographic data (age, education, marital status, employment, family members) and clinical data (previous oncological therapies, ongoing therapy, start date of therapy, follow-up in DH1, use of psychotropic drugs); the distress thermometer (DT) and its list of problems (PL). The DT is a self-report tool used to detect distress levels and associated issues; it measures distress on a scale from 0 (no distress) to 10 (severe distress). Recommended cut-off scores for identifying clinically significant distress vary according to the objectives of the screening [22,23]. Boyes [24] identified a cut-off of 4 to identify patients with a significant degree of distress. The PL consists of a list of problems that patients can tick to identify which ones contribute to increasing their level of distress. The questions asked are related to physical health (e.g., constipation, pain, fatigue), emotions (e.g., concern, sadness), possible practical problems (e.g., transportation), family problems (e.g., caring for family/children), and spiritual/existential problems. A VAS scale of 1 to 10 was also used to evaluate whether the nurse’s referral of the patient for psychology consultation was appropriate or inappropriate. The cut-off in identifying an appropriate referral was 4 (appropriate referral if the VAS > 4). This cut-off value was deemed valid for the Italian population by Grassi et al. in 2013 [25]. At the end of the training program, a satisfaction survey was issued to the nurses to measure the quality of the training received.

### 2.4. Phases of the Study

Phase 1: Training: In the first phase, the DH1 nursing staff participating in the study were randomly divided into two groups, one to be trained and another that would not be trained. The training course was developed through an integration of theoretical knowledge, experiential learning techniques, and evidence-based assessment tools to enhance nurses’ skills in recognizing and responding to emotional distress in oncology patients [25,26,27]. Group F was made up of nurses “trained” in communication and relational skills needed to properly detect emotional distress in cancer patients. Group NF comprised “untrained” nurses, i.e., those who did not receive the training. The training for Group F was conducted by two qualified psycho–oncologists, who followed a theoretical and experiential methodology. The course was made up of two parts, a theoretical part lasting 3 h and an experiential part consisting of 3 training sessions lasting 2 h each. The theoretical part included an oral presentation where the trainers shared up-to-date scientific data related to the study itself. To enable active participation and communication, the information was sent to the participants 5 days in advance of the training session. The experiential part included group discussions on clinical cases, role playing, and gathering of feedback from the participants. The course was held over an entire month, on days scheduled in advance by the DH1 nursing coordinator and shared in advance with the nursing staff, both to ensure participation in the training event by those who had been selected and also to guarantee continuity of patient care. Nurses from both groups were informed about the location for the meetings with the patients and the dates planned for the data collection. They were also taught how to assess the adequacy of the patients’ scores on the distress thermometer (DT) and trouble list (PL) (Table 1).

Phase 2: Enrollment/patient management: In the second phase of the study, the nurses involved enrolled the patients who met the inclusion criteria, explaining both the objectives of the survey and how the data would be collected. The patients were asked to carefully read the informed consent form to ensure that they had all the information needed when deciding whether or not to participate in the study. The patients were randomly divided into two groups (group A and Group B); the randomized list was generated electronically and with a ratio of 1:1. Group A was made up of patients who were given the questionnaires by the trained nurses; Group B was made up of patients who were given the questionnaires by untrained nurses. Days were for the enrollment of patients were identified and indicated, ensuring the presence on shift of both the trained and non-trained nurses, so that the objectives of the study could be properly explained and to facilitate the meetings with the nurses in the dedicated room in DH1. As previously pointed out, the patients were not told whether the nurse they would be dealing with had been trained or not, to avoid this information potentially influencing them during the interview. Patients were given the DT and the socio-demographic and clinical data collection form, which they were asked to fill out in a dedicated quiet room of the DH1, far from any disturbing elements. Once the questionnaires had been filled in, the tools were collected by the nurse, who then studied the DT questionnaire and had a brief meeting with the patient (in the dedicated room) to evaluate the congruity of the score given by the patient. Then, based on their interpretation of the results, they labeled the questionnaire with either “AP” (appropriate) or “NAP” (not appropriate), to differentiate between those patients that according to them, needed to be sent for a psychological consultation or not.

Phase 3: Interview with the psycho–oncologist. In the last phase of the study, all patients, regardless of the nurse’s assessment, were interviewed by the psycho–oncologist. Each interview was scheduled based on the availability of both the patient and the psycho–oncologist. The psycho–oncologist used a VAS scale (from 1 to 10) to determine whether the referral made by the nurse was appropriate or not. The psycho–oncologists were also kept in the dark about whether or not the nurse referring the patient had been trained. Before the questionnaires were administered, each nurse and patient participating in the study were assigned identification codes to allow the researchers to trace the data collection/analysis back to the relevant groups.

### 2.5. Statistical Analysis

According to the study design methodology, descriptive statistics were used to summarize the relevant information, presented as mean and relative standard deviation (SD) for continuous variables and as frequencies and percentage values for categorical variables. The concordance between the psycho–oncologist’s opinion and the nurses’ choices regarding the referral of patients for psychological consultation was evaluated using Cohen’s K statistic (K). A *p*-value < 0.05 was considered statistically significant. Statistical analyses were performed with SPSS 29.0 software (SPSS, Chicago, IL, USA).

## 3. Results

Twenty patients were recruited, ten of whom were randomly assigned to the group of trained nurses and ten to the group of untrained nurses. Four nursing units were involved, two for each group, to limit the variability in the evaluation. The sample of patients consisted predominantly of men (60%), married (50%), with a medium-high level of education (55%), who worked as employees (35%) (Table 2).

The patients had different oncological pathologies and underwent surgical treatments (70%) as well as chemotherapy (60%) (Table 1). The patients had an average score of 5 on the DT, their emotional problems were mainly related to fear and concern (55%), nervousness (50%), and sadness (40%), and their physical problems were mainly issues of fatigue (75%), memory (40%), tingling hands and feet, and low self-esteem (35%) (Table 3).

**Figure 1:**
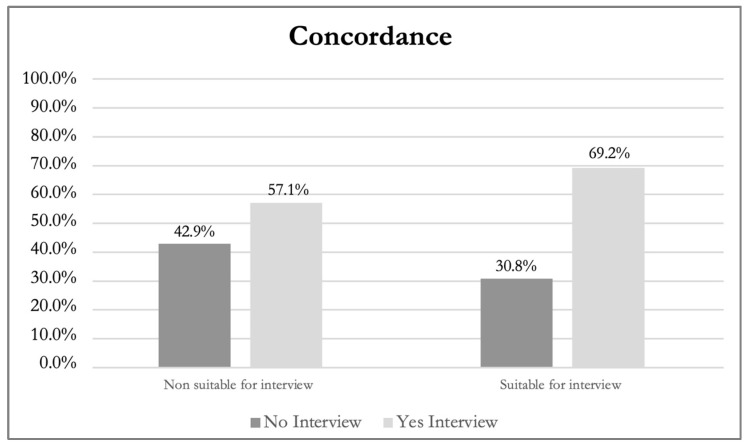
Referral Decisions by Psycho-Oncologist and Nurse.

The rough concordance between the psycho–oncologist and the nurses in identifying patients in need of psychological consultation showed that for 12 out of 20 patients examined (60%), the two professionals equally understood their real needs, indicating for 9 of them the need for a referral and no referral for the 3 others. Despite this, Cohen’s K statistic indicated poor overall concordance (K = 0.12); this finding was not statistically significant (*p* = 0.539) (Table 4, Figure 1).

**Figure 2:**
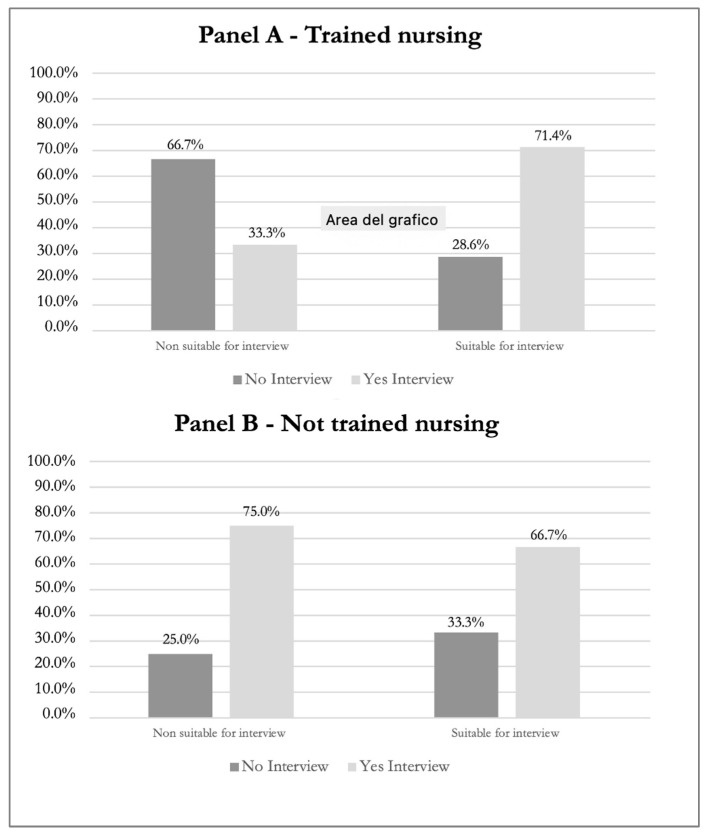
Concordance Rates of Trained vs. Untrained nurses.

We evaluated the concordance by overlaying the training status of the nurses. For the trained nurses, we observed that for seven patients out of ten (70%), the two professionals identified their real needs in the same way; however, the concordance was only slight, with a K value equal to 0.35, which was not statistically significant (*p* = 0.260) (Table 5, Figure 2). Although the Kappa value suggested some agreement, the *p*-value indicated that this agreement was not statistically significant.

Finally, with regard to the group of untrained nurses, for only five of the patients (50%) did the two professionals agree on their need for a psychological consultation. The concordance assessed with Kappa (K) was poor, with a value of −0.09, indicating weak agreement between the professionals (*p* = 0.778). This *p*-value indicated that the observed concordance was not statistically significant, reinforcing that there was no reliable agreement on the necessity of psychological consultation among the nurses assessed (Table 6, Figure 2).

In general, although the K value was never statistically significant and showed a weak overall concordance, our findings seemed to indicate that the introduction of a training course for nurses generated greater awareness of the needs of patients, optimizing the support service provided by the psycho–oncologist, given that they would only be sent those who were really in need of consultation.

The nurses participating in the training expressed a high level of satisfaction (Table 7).

## 4. Discussion

The objective of this study was to assess the impact of the training of nursing staff in the detection of distress and the appropriateness of patients’ referral for psycho–oncological consultation. Nurses are uniquely positioned to detect psychological distress, due to their constant and direct interaction with patients throughout the treatment journey [28]. Their access and established relationships allow them to observe subtle changes in patients’ emotional states that other healthcare professionals might miss. This intrinsic access is critical, as early identification can significantly impact patient outcomes [29].

Furthermore, addressing these psycho-social needs through trained nursing practice not only enhances patient care but also reduces long-term healthcare costs. Therefore, investing in specialized training for nurses not only improves their ability to recognize and address distress but also positions them as essential players in reducing healthcare costs at a systemic level [30]. However, nurses often experience discomfort when treating psycho-social issues because they do not know how to deal with these topics with patients [31]. Previous studies have shown that specific and ongoing training of health workers improves the rate of success of screening [13,32,33,34,35]. The results of our pilot study suggest that specialized training can ensure appropriate referral of patients for psycho–oncological consults. The greater success rate for trained nurses versus untrained nurses when referring patients for consultation confirms the effectiveness of training in improving the skills and awareness of health workers. Early and appropriate detection of distress can reduce levels of anxiety in cancer patients as well as the overall costs of health care [36,37]; it can also improve adherence to chemotherapy treatment [38] and hormone therapy [39], enhance quality of life [40], and increase survival time [41]. Targeted training becomes the key element in acquiring the skills necessary for the screening of distress, to enable nurses to know what to look for when referring patients for psycho–oncology consultation when appropriate. The results of our study also showed that the participating nurses expressed a high level of satisfaction with the training program itself. This can be attributed to the fact that the content of the training met the needs of the nurses, both from a cognitive and clinical perspective, by dealing with common problems related to psychological distress in cancer patients. In addition, the experiential part of the course, which included group discussions and role playing, [42] allowed participants to share their experiences and elaborate on their emotions with the support of an experienced psycho–oncologist [43,44,45]. Patients involved in our study showed an average level of psychological distress of (5), reflecting similar results to those reported in the recent literature [46]. A significant percentage of the sample showed emotional as well as physical problems, emphasizing the influence that psycho-social issues can have on cancer treatment. The ability of nurses to make appropriate referrals could increase the number of patients referred on time for psycho–oncological consultation. The psycho-social problems of patients must be recognized and managed as early in the treatment as possible, and with nurses making the right referrals, this is possible. The DT can help nurses with this task, but implementation of this tool alone is not enough; it is essential to properly train health professionals, as demonstrated by our findings and also by other existing studies [34,47,48]. These findings suggest that training on the psychological issues affecting cancer patients can significantly improve the care they receive. However, while our study demonstrated improved referral rates for psycho–oncological consultations post-training, the concordance between training and patient outcomes was weaker than anticipated. Several factors could have contributed to this discrepancy [49]. For instance, intrinsic variables such as patients’ pre-existing mental health conditions or external factors influencing their willingness to engage with psycho–oncological services may have diluted the concordance.

Furthermore, the previous literature indicates that the effectiveness of training programs can vary widely, influenced by diverse healthcare settings and patient demographics [43]. Future research should explore quantitative outcomes and delve into qualitative dimensions to understand patient attitudes toward referrals. This nuanced approach will enhance our comprehension of the psycho-social dimensions of cancer care and the factors that facilitate or hinder appropriate referrals.

### Limitations of the Study and Future Recommendations

The pilot study has some limitations. The monocentricity and the small size of the sample make the results non-generalizable. Moreover, the absence of a control group reduces the ability to attribute results solely to the training received, particularly with regard to homogeneity between the groups before the intervention. It is important to note that this research represents preliminary exploration and that the results should be interpreted in this context. Therefore, it may be useful as a starting point for future studies.

To measure the effectiveness of training, future studies should consider the following focal points:

1. Control variables: Collection of data on additional demographic and clinical characteristics, such as socioeconomic status, cancer type, and treatment regimens, should be planned through the administration of specific questionnaires, to better assess the effects of additional variables on the patients’ status (level of suffering, quality of life, depression, specific needs, etc.);
2. Longitudinal analysis: A longitudinal analysis should be conducted to assess changes in levels of patients’ suffering over time. By assessing psychological distress at multiple points in time, the trajectory of distress and the long-term impact of nursing education on referral practices can be better accommodated. These data can provide information on the long-term effectiveness of interventions;
3. To enrich the results, other specific questionnaires could be used to assess, for example, depression, anxiety, or quality of life, in order to obtain a more complete picture of patients’ psychological health and consequently improve training programs or qualitative study designs to understand patients’ perspectives on referrals to psycho–oncology services.

Furthermore, to ensure the generalizability of our results, a multicenter study should be conducted to diversify the patient population. This would also offer insights into the implementation of training programs in different healthcare settings.

## 5. Conclusions

This pilot study highlights the essential role of targeted psycho–oncological training for nursing staff in enhancing the recognition of psychological distress and facilitating appropriate referrals for psycho–oncological consultations. The data indicate that trained nurses demonstrated greater awareness and ability to identify patients in need of psychological support, compared with their untrained counterparts. Despite the promising trends observed, we acknowledge that the statistical significance of our findings is limited. This suggests that while training may foster better patient assessment skills among nurses, further evidence is needed to firmly establish the effectiveness of such interventions. Nevertheless, the training model introduced has the potential to enrich nursing competencies and address gaps in patient evaluations and referrals. Future research, particularly randomized controlled trials, will be vital in validating these preliminary findings and investigating the long-term effects on patients’ psychological health and clinical outcomes. Additionally, engaging in qualitative studies is important in order to comprehend patient experiences regarding referrals and adherence to treatment. In conclusion, investing in psycho–oncological training for nurses is crucial not only for promoting patient well-being but also for enhancing the overall effectiveness of cancer care, ultimately leading to improved treatment experiences and outcomes for patients.

## Figures and Tables

**Table 1:** Training.

Module	Description	Duration	Activities/Methods
Introduction to Psycho–Oncology	Basic concepts of psycho–oncology, the importance of communication in oncological care.	3 h	Theoretical
Recognizing Emotional Distress	Techniques for identifying emotional distress in cancer patients, signs, and symptoms to observe.	2 h	Experiential (role playing, discussion)
Effective Communication Techniques	Tools and techniques for empathetic and supportive communication with patients.	2 h	Experiential (simulations)
Managing Difficult Situations	Strategies for dealing with crises and supporting patients and their families.	2 h	Experiential (case studies)
Implementation of Assessment Tools	Introduction to evidence-based tools for assessing emotional distress and how to use them.	2 h	Theoretical + experiential
Evaluation and Final Feedback	Final discussion on the skills acquired, evaluation of the training course, feedback from participants.	2 h	Interactive discussion

**Table 2:** Clinical and socio-demographic data.

	N	%
**Gender**		
M	12	60
F	8	40
**Education**		
Middle school	2	10
High school	11	55
Degree	6	30
Data missing	1	5
**Marital status**		
Single	3	15
Married	10	50
Divorced	5	25
Widow/widower	1	5
Data missing	1	5
**Employment**		
Employee	7	35
Unemployed	3	15
Retired	4	20
Self-employed	2	10
Merchant	1	5
Blue collar	1	5
Other	2	10
**Lives alone**		
Yes	3	15
No	17	85
**Location of the tumor**		
Ovary	4	20
Urogenital	3	15
Breast	2	10
Melanoma	2	10
Testicle	2	10
Other	7	35
**Surgery**		
Yes	14	70
No	6	30
**Chemotherapy**		
Yes	12	60
No	8	40
**Radiotherapy**		
Yes	2	10
No	18	90

**Table 3:** Distress Questionnaire.

Scale of distress (median (min–max))	5 (0–7)	
	YES (N)	(%)
**Practical Problems**		
Childcare	0	-
Housing	0	-
Economical	4	20
School/Work	3	15
Transportation	4	20
**Relationship Problems**		
Relationship with partner	0	-
Relationship with children	0	-
Relationship with others	0	-
**Emotional Problems**		
Depression	0	-
Fears	11	55
Nervousness	10	50
Sadness	8	40
Worries	11	55
Loss of desire for daily routine	1	5
**Spiritual Problems**		
Faith	2	10
**Physical Problems**		
Sleep	6	30
Pain	2	10
Washing/Dressing	4	20
Nausea	4	20
Fatigue	15	75
Problems moving around	3	15
Breathing problems	3	15
Mouth ulcers	2	10
Eating disorders	2	10
Digestion problems	2	10
Constipation	4	20
Urination disorder	2	10
Fever	0	-
Dry skin	6	30
Stuffy nose/Dryness	4	20
Tingling hands/feet	7	35
Swelling	6	30
Sexual problems	2	10
Diarrhea	2	10
Memory issues	8	40
Self esteem	7	35

**Table 4:** Concordance between psycho–oncologist’s and nurses’ assessments of patients’ need for psychological consultation.

		Interview with the Psychologist		Total
		Non-Suitable	Suitable	
Nurse	No interview	3 (42.9%)	4 (30.8%)	7 (35.0%)
	Interview	4 (57.1%)	9 (69.2%)	13 (65.0%)
Total		7 (100.0%)	13 (100.0%)	20 (100.0%)

**Table 5:** Trained Nurses.

		Interview with the Psychologist		Total
		Non-Suitable	Suitable	
Trained Nurse	No interview	2 (66.7%)	2 (28.6%)	4 (40.0%)
	Interview	1 (33.3%)	5 (71.4%)	6 (60.0%)
Total		3 (100.0%)	7 (100.0%)	10 (100.0%)

**Table 6:** Non-trained nurses.

		Interview with the Psychologist		Total
		Non-Suitable	Suitable	
Non-trained Nurses	No Interview	1 (25.0%)	2 (33.3%)	3 (30.0%)
	Interview	3 (75.0%)	4 (66.7%)	7 (70.0%)
Total		4 (100.0%)	6 (100.0%)	10 (100.0%)

**Table 7:** Mean satisfaction results for event.

	Nurses
Day one	5/5
Day two	5/5
Day three	5/5

## Data Availability

The data are not publicly shared, to protect the privacy of the participants. They are available on request from the corresponding author (F.Gambalunga).
